# Dynamic Control of Electron Transfers in Diflavin Reductases

**DOI:** 10.3390/ijms131115012

**Published:** 2012-11-15

**Authors:** Louise Aigrain, Fataneh Fatemi, Oriane Frances, Ewen Lescop, Gilles Truan

**Affiliations:** 1 Gene Machines Group, Clarendon Laboratory, Department of Physics, University of Oxford, Parks Road, Oxford OX1 3PU, UK; E-Mail: l.aigrain1@physics.ox.ac.uk; 2 Institut de Chimie des Substances Naturelles, CNRS, UPR 2301, Centre de Recherche de Gif, 1 Av. de la Terrasse, 91198 Gif-sur-Yvette Cedex, France; E-Mails: fatemi@icsn.cnrs-gif.fr (F.F.); oriane.frances@icsn.cnrs-gif.fr (O.F.); ewen.lescop@icsn.cnrs-gif.fr (E.L.); 3 Université de Toulouse; INSA, UPS, INP; LISBP, 135 Avenue de Rangueil, F-31077 Toulouse, France; 4 INRA, UMR792 Ingénierie des Systèmes Biologiques et des Procédés, F-31400 Toulouse, France; 5 CNRS, UMR5504, F-31400 Toulouse, France

**Keywords:** diflavin reductases, multidomain proteins, protein dynamics, NOS, CPR, Cytochrome P450 reductase, MSR, NR1, SiR

## Abstract

Diflavin reductases are essential proteins capable of splitting the two-electron flux from reduced pyridine nucleotides to a variety of one electron acceptors. The primary sequence of diflavin reductases shows a conserved domain organization harboring two catalytic domains bound to the FAD and FMN flavins sandwiched by one or several non-catalytic domains. The catalytic domains are analogous to existing globular proteins: the FMN domain is analogous to flavodoxins while the FAD domain resembles ferredoxin reductases. The first structural determination of one member of the diflavin reductases family raised some questions about the architecture of the enzyme during catalysis: both FMN and FAD were in perfect position for interflavin transfers but the steric hindrance of the FAD domain rapidly prompted more complex hypotheses on the possible mechanisms for the electron transfer from FMN to external acceptors. Hypotheses of domain reorganization during catalysis in the context of the different members of this family were given by many groups during the past twenty years. This review will address the recent advances in various structural approaches that have highlighted specific dynamic features of diflavin reductases.

## 1. Introduction

### 1.1. Generalities

Flavoproteins represent 1%–3% of all proteins present in prokaryotic and eukaryotic genomes [1,2] and about half of the proteins involved in electron transfer [3]. They are central in redox processes and their originality comes from the particular properties of their flavinic cofactors that exist in three different redox states (oxidized, semiquinone and hydroquinone) and can thereby catalyze both oneand two-electron transfer (ET) reactions. The two flavins in diflavin reductase proteins (FAD and FMN) are able to split the dielectronic flux from the reduced pyridine nucleotides, either NADH or NADPH, to a variety of mono-electronic heme protein acceptors [4]. The electron flux usually follows the linear pathway NAD(P)H → FAD → FMN → final acceptor. While FAD is responsible for the electron partition, the FMN prosthetic group functions as a shuttle between the FAD and the prosthetic group of the acceptor.

This diflavin reductases family includes the NADPH-cytochrome P450 reductase (CPR, EC 1.6.2.4), the flavoprotein subunits of bacterial sulfite reductase (SiR, EC 1.8.99.3) and of the mammalian methionine synthase reductase (MSR, EC 1.16.1.18), the reductase part of the nitric oxide synthase (NOS, EC 1.14.13.39) and of cytochrome P450-BM3 (BM3, EC 1.14.14.1) and the novel reductase 1 cytoplasmic protein (NR1).

The CPR enzyme was the first diflavin reductase to be identified [5] and was initially characterized as a cytochrome *c* (cyt *c*) reductase [6,7]. The flavinic nature of the prosthetic groups was first determined as two FAD molecules [8,9]. As purification techniques improved, the exact cofactor content was determined. Furthermore, the cellular function of CPR as an electron carrier for microsomal cytochrome P450 was revealed [10–13]. BM3 was first identified from the cytosol of *Bacillus megaterium* as the enzyme capable of hydroxylating saturated fatty acids [14] and represented the first known autonomous P450 system [15]. MSR was discovered following analysis of patients suffering from a reduced level of methionine synthase activity (cblE disease) correlated with a defect in a NADPH-linked reducing system [16]. MSR was cloned, expressed and assigned to the methionine synthase electron carrier using consensus sequences that predicted the binding sites for FMN, FAD, and NADPH [17,18]. SiR was identified as a redox partner of the enzymatic complex devoted to the biosynthesis of l-cystein from sulfate [19]. SiR is active as an octamer, each monomer bearing one molecule of FMN and FAD [20–22]. The discovery of NOS followed the identification of NO as an essential regulator in mammalian biology and not only a poison or a pollutant. Three isoenzymes catalyzing the regulated oxidation of l-arginine to NO and l-citrulline in mammals were identified: endothelial NOS (eNOS), inducible NOS (iNOS) and neuronal NOS (nNOS) [23–26]. nNOS and eNOS are reversibly activated by the Ca^2+^ binding protein calmodulin (CaM), enabling their participation in biological signaling cascades. By contrast, iNOS binds CaM regardless of Ca^2+^ concentration and remains constitutively active in absence of calcium [27,28]. Finally, the most recently discovered diflavin reductase is NR1 [29]. Although its physiological role remains unclear, NR1 appears widely overexpressed in human cancer cell lines [29]. More recently, Varadarajan *et al.* discovered that the primary sequence of NR1 was close to that of ATR3 [30] that deviates from the strictly conserved FMN, FAD and NADPH-binding domains present in all CPR. Furthermore, ATR3 did not sustain human P450 1A2 activity in contrast with most CPR [30]. A second study showed that NR1 can substitute MSR and fully activate the methionine synthase *in vitro* in the presence of cytochrome *b*_5_. However, the *in vivo* function of NR1 remains uncertain due to the lack of information concerning its expression levels in cells [31].

### 1.2. Domain Architecture

The earliest characterization of diflavin reductase architecture came from the examination of the primary sequences and gave a first indication of the domain arrangement and interactions. Comparisons of primary structures of CPR, SiR, MSR or NOS evidenced two distinct and specific binding sites for FMN and FAD [32,33] and a good conservation between the FMN domain or the FAD domain in diflavin reductases with their probable ancestors, a flavodoxin and a ferredoxin reductase respectively [34,35]. Prior to any high resolution structural information, the analysis of isolated FMN or FAD domains, obtained either by independent expression [36] or by proteolysis [37], confirmed the functions associated with each domain and the electronic pathways through CPR [36–41]. The FAD domains isolated from CPR [36,41] or from P450 BM3 [37,42] were shown to support the reduction of monoelectronic acceptors such as ferricyanide while the FMN domain turned out to sustain the reduction of cyt *c* and was thus supposed to be the domain capable of ET to the natural partners.

The depletion of the FMN cofactor in full-length diflavin reductases, either via specific flavin removal [43] or by site-directed mutagenesis in the FMN binding site [44–47], has also been used to decipher the role of each flavin in the overall ET mechanism. Mutants of MSR (A129T [45]), BM3 (cluster Y536-G570-W574 [46]) and nNOS (cluster F892-D918-E919 [44]) are fully depleted from their FMN cofactor. Their activities toward ferricyanide were globally well preserved but they exhibited very low or even fully abrogated ET efficiency toward cyt *c* or BM3 heme domain. This was the definitive proof that the FMN domain was the last electron-carrier element prior to the final ET and was therefore capable of interacting with both the FAD domains and the various heme partners [46].

It is currently accepted that diflavin reductases result from the fusion between two ancestral genes encoding for a ferredoxin reductase protein and a flavodoxin protein [35,48]. Analysis of the primary structures of diflavin reductases revealed the existence of a third domain, referred thereafter as “connecting domain”, sandwiched between the two flavin domains which was later confirmed by the first tertiary structure of a diflavin reductase [49]. This domain shares no homology with other protein structures present in the Protein Data Bank (PDB) [50]. However, this connecting domain possesses well-defined secondary structure elements (7 α helices) as opposed to single loop containing flexible linkers lacking stable secondary or tertiary conformation.

**Figure 1:**
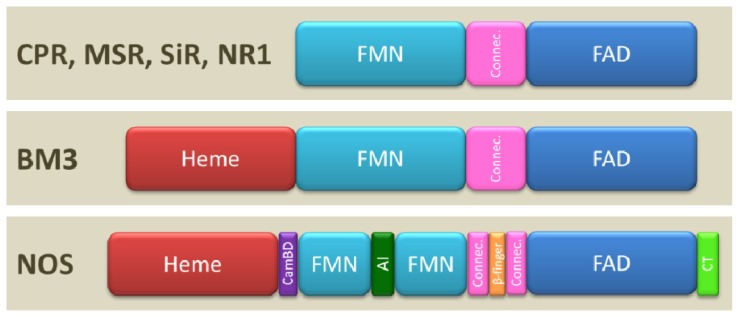
Architecture of the structural domains present in diflavin reductases. Three domains are common to all diflavin reductases: the FMN domain (light blue), analogous to flavodoxin, the FAD domain (dark blue), analogous to ferredoxin reductase, and the connecting domain (Connec., pink) whose fold is unique to diflavin reductases. The reductase part of BM3 and nitric oxide synthase (NOS) are fused to their final acceptor, the heme domain (in red). NOS also presents structural elements absent from the other diflavin reductases and that are implicated in the regulation of electron flow through the protein: a calmodulin binding domain (CamBD, purple) inserted between the heme and FMN domains, a sequence of β-fingers (orange) in the connecting domain. In eNOS and nNOS an autoinhibitory insert (AI, dark green) in the middle of the FMN domain and a *C*-terminal tail (CT, light green) are also present.

The CPR, MSR, SiR and NR1 all possess the minimal elements of the diflavin reductases protein architecture including 3 distinct domains (FAD, FMN and connecting domains, Figure 1). However, the CPR is the only diflavin reductase to possess a 50–60 residue *N*-terminal membrane anchor for binding at the endoplasmic reticulum membrane. In contrast, the other members of the family are soluble. A second difference resides in the size of the flexible loop (linker) joining the connecting and the FMN domains which is longer in MSR compared to other diflavin reductases (80 residues in MSR compared to 10–15 residues in other diflavin reductases). In the cases of BM3 and NOS, the diflavin reductase part is fused to its final acceptor, the heme domain. NOS displays the highest level of architecture complexity and possesses three to four additional regulatory elements between and/or within its domains: a CaM binding domain between the FMN and heme domains, a β-finger fold between the FAD and NADPH binding sites and, for eNOS and nNOS, a 42–45 residue autoinhibitory insert (AI) in the middle of the FMN domain and a 21–42 residue *C*-terminal tail (CT) (Figure 1). These elements and their regulation mode were described in the literature [28,51]. Finally, some molecular systems such as NOS, BM3 and MSR are also prone to oligomerization, which is of functional importance (*vide infra*).

### 1.3. Oligomerization

**Figure 2:**
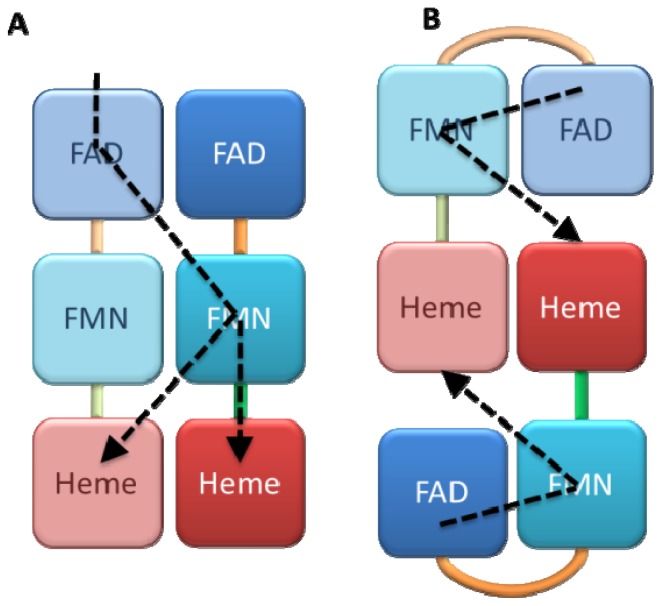
Electron transfer (ET) through P450 BM3 and NOS dimers. In the scheme **A**, interflavin ET occurs between FAD and FMN domains from distinct molecules and the final transfer may be either intra- or intermolecular. In the scheme **B**, the interflavin ET occurs within the same molecule and the FMN to heme ET is intermolecular. NOS functions only with scheme **B** whereas BM3 might adopt both **A** and **B** schemes.

Sedimentation measurements and size exclusion chromatography of P450 BM3 demonstrated the existence of a stable dimeric form of the enzyme [52]. Moreover, P450 BM3 catalytic activity felt dramatically at very low concentration of the enzyme, indicative of the dissociation of the active dimer into an inactive monomer. However, the interflavin electron transfer (ET) was not affected by dilution, which suggested an intramolecular interflavin ET transfer [47]. These results support the hypothesis of a first ET between FAD and FMN that would take place within the same monomer unit while ET between FMN and heme domains would involve both subunits of the dimer (Figure 2B). Using site directed mutagenesis of P450 BM3, Kitazume *et al.* demonstrated that the activity of dimers remained unchanged when interflavin ET within each monomer unit was blocked while intermolecular ET was still possible [53]. In this case, the interflavin ET would rather take place between the two different units of a dimer while heme domains could accept electrons from both of reductase parts of the dimer (Figure 2A). Clearly other experiments are needed to exactly determine what the active forms of P450 BM3 are. Alternatively, both pathways may coexist with different efficiencies and the possibility that interflavin ET between subunits may complement intrasubunit transfer when this latter is not possible has not yet been investigated.

Like BM3, NOS forms stable dimers that are required for its activity [54]. Biochemical studies using mutagenesis and protein engineering have demonstrated that part of the electronic pathway is effective through the homodimer. During NOS catalysis, ET from FAD to FMN domains occurs within one monomer (intramolecular) while the transfer from FMN to heme proceeds through the homodimer via an intersubunit ET (tethered model: Figure 2B) [55,56].

Some diflavin reductases forming oligomers present the particularity of providing more than two electrons to their final acceptors. The dimeric active form of NOS transfers six electrons from three NADPH molecules to two heme molecules, each of them requiring three electrons for the synthesis of two NO molecules. Similarly, SiR forms octamers that provide the twenty-four electrons required for the formation of sulfite in the catalytic site of the acceptor. It is still unknown whether or not oligomerization allows the heme domains to receive electrons from different reductase domains and whether inter-reductase ET avoids the accumulation of ROS when the concentration of NADPH is low [4].

### 1.4. Modular Assembly of Domains

The evolution of diflavin reductase genes probably reflects the optimization of their electron carrier efficiency in heterogeneous systems. The conservation of several structural characteristics, principally the order of domains and the cluster of residues responsible for cofactor binding, highlights the importance of the global architecture to achieve the ET function in diflavin reductases. On the contrary, the particularities developed in some members of the family (regulatory elements, oligomerization or fusion with final acceptors) demonstrate the evolvability of these enzymes to adopt new functions or more tightly controlled regulatory mechanisms.

Like most multidomain proteins, properties of diflavin reductases represent more than the sum of properties of the isolated domains [57–59]. Indeed, although each flavin domain is able to fold independently, the reconstitution of the ET properties with isolated domains is either impossible or particularly inefficient [36]. For example, the cyt *c* reductase activity of reconstituted human CPR from the isolated FMN and FAD domains is only about 2% of its native activity [36]. Modules assembly into multidomain proteins is consequently not free of incidence. Such fusions offer advantages in terms of partner promiscuity and cellular colocalization as well as by reducing the entropic cost to bring the two partners in close distance during the catalytic cycle (high local concentration). But, more importantly, an intrinsic property of the multidomain assembly of diflavin reductases is the optimization of interactions with several partners through the control of domain orientation [60,61]. The proper global architecture and orientation are probably mediated by the linker or connecting domains joining the catalytic domains [60,62–65]. The drastic decrease of ET efficiency to acceptors in reconstituted system evidences the importance of the connecting and linker domains between the FMN and FAD domains as well as other regulating elements present in more complex systems (NOS and BM3).

CaM binding controls the various ET (FAD to FMN and FMN to heme) in NOS [27,66,67] but other regulatory elements also control NOS activity (AI or CT, Figure 1). A comparison of the three NOS isoforms, using native or truncated forms (lacking the regulatory elements), with other diflavin reductases shed some insights on the function of these regulatory elements before any structural information was obtained. For example, the presence of the AI element inversely correlates with the rate of NO synthesis or cyt *c* reduction in the various NOS isoforms. Consequently, nNOS and eNOS synthesize NO at rates approximately 1/3 and 1/20 that of iNOS which does not possess any AI at all [28,68]. Deletion of AI from the nNOS or eNOS removes the CaM binding requirement for NOS activity and destabilizes FMN binding [69,70]. The AI element may thus serve to enhance (in association with the CaM binding domain) the regulatory effect on ET efficiency between FMN and heme [28]. In addition, NOS mutants in which the CT element was partially or fully deleted exhibit a 5–10 times greater rate of cyt *c* reduction and 20% higher NO synthesis than native isoforms [71,72]. In these mutants, the ET rate reaches the values observed with CPR (which naturally lacks AI) [28]. In summary, the CT appears to be the main dominant negative regulator of interflavin ET in absence of CaM binding while AI behaves as the main dominant negative element for ET regulation in presence of CaM binding.

These different results clearly identify a modular assembly of the various non-catalytic domains in diflavin reductases. When present, these additional motifs have unambiguous regulatory roles in adjusting the catalytic ET efficiency in response to external signaling. However, the detailed mechanism of the regulatory events leading to enhanced or reduced ET was highly elusive until high resolution structures of diflavin reductase became available.

## 2. First Evidences of Interdomain Dynamics

### 2.1. Crystallography

**Figure 3:**
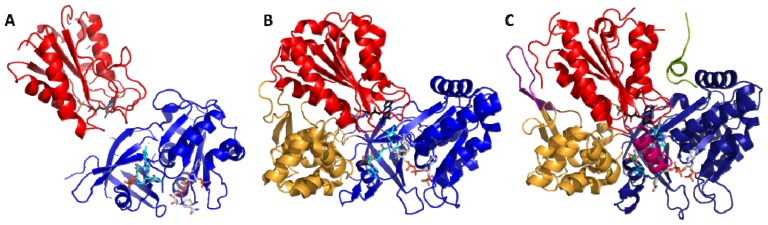
Crystallographic structures of diflavin reductases and of their ancestor proteins. (**A**) Structures of a flavodoxin (PDB ID: 3KPA) and a ferredoxin reductase (PDB ID: 2VNH). (**B**) Structure of the rat CPR (PDB ID: 1AMO). (**C**) Structure of the rat eNOS (PDB ID: 1TLL). FAD and FMN domains and their analogous ferredoxin reductase and flavodoxin are colored respectively in blue and red. The flavodoxin and ferredoxin reductase are shown in panel **A** with the same orientation as the FMN and FAD domains respectively in panels **B** and **C**. The connecting domains are colored in yellow and NOS regulatory elements AI, CT and β-fingers are represented respectively in green, pink and purple colors. FAD, FMN and NADP^+^ molecules are depicted in stick colored in cyan, black and orange respectively.

The crystallographic structure of the rat CPR by Wang *et al.* in 1997 corroborated the modular nature of the system constituted of independently folded domains [49]. In this structure, the similarity of the FAD and FMN domains with ferredoxin reductase and flavodoxin folds was obvious (Figure 3A,B). The FAD and connecting domains fold as independent globular domains although their primary sequences are intimately intertwined. In this structure, the connecting domain seemed to hold both flavin domains in close contact so that they are well aligned and in close vicinity. The distance between FAD and FMN (less than 4 Å) and their orientation is compatible with a rapid internal ET. However, in this conformation, the FMN cofactor is buried so deeply inside the CPR that no ET to external acceptors seems possible and accordingly, this structure is often referred as the closed conformation. Docking experiments with several P450 structures showed that the minimal FMN-heme distance that could be reached upon docking (>20 Å) is not compatible with a direct ET. Therefore, the scientific community hypothesized that CPR (and by extension diflavin reductases) could adopt other conformations showing an unrestricted access of the FMN to external acceptors for optimal intermolecular ET.

Over the years several additional crystallographic structures were obtained for isolated FMN or FAD domains from BM3 [73,74], the FMN domain of human CPR [75], the flavodoxin-like domain of SiR [76], the isolated FAD domains from nNOS and MSR [77,78]. All these structures superimposed well with the equivalent domains in the structure of the native form of the rat CPR and therefore did not bring any further information about the possible alternative orientations of the domains. Full length reductases were also crystallized (native or mutant nNOS reductase [51], human [79], yeast [80] and mutant rat CPR [81]) but their conformations remained very similar to the structure of the native form of rat CPR (for example the overall rms deviation for the backbone atoms between yeast CPR or nNOS and rat CPR is 1.44 and 0.6 Å respectively, Figure 3C). Notably, the relative orientation and positioning of the FAD and connecting domains is very well conserved in all available CPR structures, and, as a whole, they are sometimes considered as a single rigid FAD/connecting domain.

However, structural discrepancies reinforced the hypothesis of the existence of alternate conformations. For example, the BM3 FMN domain co-crystallized with the heme domain and, despite proteolysis of the linker between both protein parts, the resulting domains assembly was consistent with a FMN → heme ET [73]. A structural alignment of the FMN domain in the latter structure with the equivalent FMN domain from the rat or yeast CPR leads to a steric clash between the BM3 heme domain and FAD/connecting domains, again endorsing the idea of the necessity of domains reorganization enabling the FMN domain to move out of the FAD domain pocket in order to interact with external acceptors.

In the three-dimensional structure of SiR [50], the electronic density of the FMN domain could not be correctly identified despite the integrity of the protein in the crystal, as verified by SDS PAGE and enzymatic activity. Docking experiments could model the position of the FMN domain in a large empty cavity in the crystal packing. The lack of electronic density, likely reflecting the mobility of this entire domain, corroborated once more the hypothesis of possible FMN domain motions in diflavin reductases. An analogous phenomenon was also observed in the nNOS three-dimensional structure [51] for which the crystallographic unit cell contains two NOS molecules (dimer) and their FMN domains exhibits 4° rotation relative to each other, a value clearly higher than the average temperature factors. Furthermore, the electron density for the connecting domain is also ill-defined.

The last structural clue was provided by the structure of the MSR FAD/connecting domains [77]. The longer linker joining the connecting and FMN domains in MSR may provide a greater degree of freedom to the FMN domain [82]. In the partial structure of MSR, the linker orientation is clearly different from the one of the closed form of CPR, suggesting that the FMN domain position could have been different if the crystallized protein had been full-length [77].

In summary, all of these different structures of domains or full-length diflavin reductases did not evidence any significant conformational variation compared to the first crystallographic structure of CPR yet the above-quoted inconsistencies constituted further strong arguments in favor of alternate conformations. Several pieces of elements point to the possibility for significant intrinsic interdomain flexibility that is manifest for instance in SiR and for which a meaningful assessment is difficult in the crystal environment. The attempts to trap alternate conformations on the wild-type enzymes may also have been rendered more difficult due their probable short lifetime during the catalytic cycle. Although the structures of different complexes with substrates or of site-directed mutants have already been determined, all of them were in the oxidized state for technical reasons, which seems so far not to be sufficient to stabilize other conformations.

### 2.2. Alternate Hypothesis

An alternate model countering the putative mechanism involving domain reorganizations during catalysis was proposed by Lamb *et al.*[80]. Yeast CPR crystallized in a conformation analogous to the rat CPR, but the authors identified a second FMN binding site. The stoichiometry of one FMN molecule per CPR was globally conserved but the occupancy of the FMN molecule was half in the regular binding site and half in an alternative site. This second site of FMN binding is positioned at the FMN and connecting domains interface, thus rendering the flavin much more accessible to the solvent. A putative derived mechanism was proposed by the authors: the shuttle function of FMN could be performed with the displacement of the cofactor itself between the regular and the more exposed site and thus does not invoke domain motion. In this model, the FMN cofactor would then receive the electrons one by one from the FAD cofactor and change from the neutral semiquinone to the putative anionic semiquinone upon switching to the second binding site, in a position ready for ET to external acceptors. Such similar rotations of the flavin cofactor exist in the *p*-hydroxybenzoate hydroxylase in which the FAD isoalloxazine ring undergoes a 33° rotation along the ribitol tail from a so-called IN (protected) to an OUT (solvent exposed) conformations upon substrate binding allowing the reduction of the flavin cofactor by NADPH only in the presence of a substrate in the active site [83]. The first “classic” positioning would allow the interflavin ET while the second would correspond to the FMN → P450 ET. More recent data supported the existence of this second binding site in yeast CPR via SPR studies [84]. This study revealed that both FAD and FMN can bind (1:1 ratio) to immobilized holo CPR with quite high affinities (around 0.3 μM for FMN and around 2–10 μM for FAD). These results cannot explain why, with such a strong calculated affinity between FMN and the second putative binding site, purified yeast CPR contains only one FMN per monomer [85]. The case of this second FMN binding site remains elusive as multiple FMN binding sites have not been observed in any other diflavin reductases and particularly in mammalian enzymes. It is therefore clear that more experiments will be needed to understand the possible role of this putative second FMN binding site and the generalization of its presence in other CPR.

## 3. Biochemical Characterization of Dynamical Behaviors

In the next paragraphs, we will present how the various biochemical characterizations of diflavin reductases support the idea of domain motions [86–88] and describe the elements or factors that may regulate them [28,89]. Some of these results were published before the initial “dynamical hypothesis” and very few papers have proposed to reunite the accumulated knowledge on the different diflavin reductases [4,90,91]. This review will therefore focus on the convergence between the dynamical aspects of the various diflavin reductases and the more recent discoveries of variations in the geometry of the domains.

### 3.1. NOS

NOS were the first diflavin reductases for which biochemical analysis strongly supported the hypothesis of relative motions of the FMN domain between the FAD and the heme domains. The first indications came from the fluorescence analysis of nNOS flavins by Brunner *et al.*[92]. In this paper, the authors discovered the existence of alternate conformations characterized by different flavin environments. These conformations did not interconvert on the fluorescence time scale and the relative populations were modified by the binding of cofactors such as CaM/Ca^2+^ or by the presence of the heme domain on the polypeptide. As the FMN was assumed to be rigidly bound to the protein due to the stacking of aromatic residues, the authors interpreted the changes in rotational relaxation and fluorescence lifetime as a reorganization of the entire FMN domain leading to a greater exposition of FMN in the CaM/Ca^2+^-free nNOS [67]. The role of CaM/Ca^2+^ binding was further characterized and turned out to be mandatory in promoting conformational changes that enhance interflavin and FMN → heme ET through nNOS [93,94] where the latter ET constitutes the limiting step of the system [95]. However, the scale of the reorganization phenomena, which might be at the level of residues, clusters or domains, remained difficult to estimate.

Other regulatory elements (AI and CT) seem also involved in NOS structural changes. In the absence of CaM, the positioning of the AI is predicted to obstruct CaM binding and enzyme activation. As a result, higher Ca^2+^ concentrations are required to displace AI and activate electron flow through eNOS and nNOS when compared to iNOS [28,51]. These structural modifications were characterized by changes in the accessibility of defined protein sites to proteolysis [68] and were consistent with the above-cited fluorescence analysis. The CT element, which lies at the *C*-terminus of NOS, is also a modulator of the ET rates as CT-truncated iNOS transfers electrons faster compared to the intact protein [71,72]. These results lead to the hypothesis that CT was able to modulate the distance and/or angle between the flavins.

**Figure 4:**
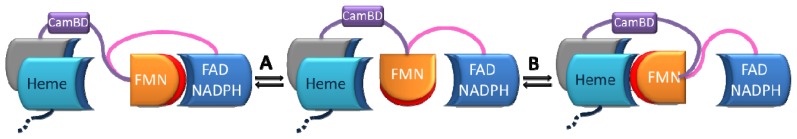
The three-state model and two equilibria describing NOS FMN domain movements during ET. On the left, the shielded conformation in which the FMN domain is awaiting for electrons from the FAD domain of the same subunit. On the right, the deshielded conformation in which the FMN domain is well positioned to transfer electrons to the heme domain of the other subunit. CaM binding increases the rates of cyt *c* reduction by moving the equilibrium B sharply to the right [87,96]. The same electronegative face of the FMN domain (red) is assumed to interact with both electropositive specific surfaces of its redox partners (blue) and the FMN domain of one NOS subunit interacts exclusively with the heme domain of the other subunit in a NOS dimer [96].

Globally, in the absence of CaM, the NOS reductase domain crystallized in the same geometry as in wild-type oxidized CPR [51], and thus adopted the closed conformation, also called FMN-shielded or electron-accepting conformation in the context of NOS, where both flavins lie in close proximity. When CaM binds, the FMN domain seems to dissociate from the FAD domain and starts interacting with the heme domain to yield the so-called FMN-deshielded or electron-donating conformations [28,96,97] (see Figure 4). In the case of nNOS, the conformational change upon CaM binding is known to involve the CT regulatory domain [98]. In absence of CaM, the CT domain stabilizes the FAD and FMN domains in the closed conformation and CaM binding relieves these long-range contacts [98]. Investigations on NOS isoforms activities in several conditions (with/without CaM, single/multiple turnover analysis…) revealed a 10-fold difference in the equilibrium between shielded and deshielded conformation that could explain the approximate 10-fold higher NO synthase and cyt *c* reductase activities of the nNOS compared to eNOS [97]. Data analysis suggests that the CaM-free NOS activities could be limited by a conformational motion of the FMN domain and more precisely by its dissociation from the FAD domain [97,99,100] while other reaction steps such as interflavin ET or NADP^+^ dissociation might constitute rate limiting steps in the CaM-bound enzymes [101,102].

To test the validity of the domain motion model, Welland *et al.*[103] investigated the role of one of the only two electrostatic contacts between the FMN and FAD domains identified in the NOS reductase structure. The charge-reversal mutation R1229E in the FAD domain of the rat nNOS logically resulted in a widening of the distance between the FMN and FAD domains, a stabilization of the deshielded form and had indeed dramatic effects on catalysis. Indeed, this mutant had enhanced NADPH → FAD hydride transfer and FMN → cyt *c* ET in the CaM-free enzyme and inhibited interflavin ET regardless of CaM presence. This result was consistent with a model in which both activated transfers occur in the deshielded form while the inactivated transfer occurs in the shielded (closed) form of the NOS.

Recently, the same authors proposed a model linking the amplitudes of the FAD reduction rate and the proportions of enzyme known to exist in the deshielded and shielded conformations [87]. The NADPH → FAD hydride transfer is a biphasic event in NOS, and for most diflavin reductases [104–106]. For the CaM-bound from of nNOS, the FAD reduction occurs with a large fast phase constituting 80% of the absorbance change, whereas this rapid phase constitutes only 20% of the absorbance change in the CaM-free enzyme. The large slow phase in this latter case has a rate constant of 6.5 s^−1^, a value close to the steady-state catalytic rate constant of cyt *c* reduction (<10 s^−1^[107,108]) and could therefore constitute the overall rate-limiting step. In addition, increasing ionic strength have similar effects than CaM binding (*i.e.*, deshielding), which might be understood by the disruption of electrostatic interactions between the FAD and FMN domains which is locked in the shielded form and therefore inhibited cyt *c* reduction. Moreover, the biphasic nature of the reduction of CaM-free nNOS by NADPH turned out to be also ionic strength dependent and could not therefore be caused by partial degradation or reoxidation of the sample. Using a series of amino acid deletions or insertions in the linker between the FMN and FAD domains, Haque *et al.* evidenced how the length of this small region controls ET between flavins and between FMN and heme [109,110]. Evidently, the control of ET by the linker length further strengthens the hypotheses of dynamical exchange between the shielded and deshielded forms in NOS.

In absence of any crystallographic structure of the deshielded conformation, a truncated NOS forms corresponding to the heme and FMN domains of the protein but no FAD domain was used to get a better insight of the FMN-heme ET and of the interaction between both domains [94,111,112]. Such constructs exhibit ET kinetics one order of magnitude higher than their holoenzymes suggesting that the ET rate limiting step in wild type NOS is indeed the conversion between the shielded and deshielded states under CaM control. A well detailed review focusing on the ET between both FMN and heme domains has been recently published by Feng [56].

All these results confirm the existence, in solution, of different conformations having slow interconversion rates [87] and led to a model (Figure 4) in which (i) the FMN domain physically performs shuttle movements between the FAD and heme domains and (ii) the frequency of these movements and the equilibrium between different conformational subpopulations depends on CaM binding and NOS isoforms [87,96].

### 3.2. CPR

Besides the incompatibility of external FMN → heme ET in the crystallographic structures of rat and yeast CPR, a second discrepancy arose from the fact that residues likely involved in CPR-cytochrome P450 recognition are also involved in FMN-FAD domain interfaces in the closed structure of CPR [73,113–117]. If these residues contact the cytochrome P450 during ET and the FAD domain in the crystallographic structure, then a separation of both flavinic domains during the catalytic cycle of CPR is clearly needed. No significant activity was reconstituted upon mixing the individual catalytic domains (leaving the connecting domain fused to the FAD domain) [36], proving that the domain assembly is mandatory to control recognitions, movements and/or positioning of the catalytic domains.

In 2011, Xia *et al.*[118] engineered two cysteine residues in the human CPR at the positions of Asp147 and Arg514 located on the FMN and FAD domains respectively and in close proximity in the wild-type human crystallographic structure [79]. The crystal structure of the mutant confirmed that the interdomain disulfide bond could be formed. The formation of the disulfide bridge added structural constraints as evidenced by the displacement of the FMN domain and the reorientation of the FMN moiety. Under these conditions, the steady-state turnover rates of reduction of cyt *c* and P450 were severely reduced compared to the wild-type enzyme. However, cysteine reduction induced by DTT treatment restored the ability of CPR to transfer electrons to its redox partners. Although the structural features of the closed form with the disulfide bridge was not equivalent to the wild-type closed form, the full restoration of CPR ET capability when the two domains were no longer attached let the authors conclude on the obligatory domain motion between the catalytic domains for efficient ET to external acceptors.

Using stopped-flow spectrometry, Gutierrez *et al.* published a series of articles concerning the different ET rates occurring in CPR [104,119–121]. First, the discrepancy between the predicted interflavin ET rate (≈10^10^ s^−1^, based on the ≈4 Å separation distance in the native structure) and the experimental measurement (55 s^−1^) revealed that this interflavin ET is most probably gated. The absence of isotope effects on reaction rates ruled out a rate-limiting deprotonation reaction of flavin semiquinones. On the other hand, an increase in the solvent viscosity, unfavorable for molecular mobility, compromised strongly the FAD → FMN ET, proving thereby the entailed mobility of catalytic domains for CPR activity.

As in NOS, the linker domain, a very flexible loop (high B factor values in all crystallographic structures) joining the FMN domain to the *N*-terminal extremity of the connecting domain, seemed also involved in allowing or even controlling catalytic domains motion. The deletion of several residues in the human CPR linker or their replacement by polyprolines (enhanced rigidity of the loop) resulted in a decrease in NADPH affinity and a 50-fold decrease in interflavin ET and cyt *c* reduction activity [122]. Such modifications, albeit located more than 60 Å away from the NADPH binding site, shed light on the crucial importance of the linker for CPR function.

Taken together, biochemical characterizations of wild-type and mutant CPR seamlessly pointed out the necessity for a structural model in which the domains organization in different oxidation states would differ from the classical structural form of wild-type oxidized CPR.

### 3.3. Chimeric Diflavin Reductases

Taking BM3 as a model, numerous studies focused on the optimization of P450 activities by building chimeric systems harboring the reductase parts of NOS or BM3 or the full CPR fused to a particular P450 [123–128]. However, very few studies addressed the specific role of each domain and their dynamical behaviors. Nonetheless, chimeric systems composed of the domains originating from the three different isoforms of mammalian NOS have been constructed and analyzed [25,110,129]. These enzymes present the advantage of preserving the integrity and fold of individual domains while allowing a global change of the interdomain interface. These works provided further insights onto the roles of catalytic or regulatory domains, their interdependency and how they may impact the catalytic cycles of the different NOS isoforms (Table 1).

Other chimeras, combining domains from nNOS and rat CPR were constructed (Table 2) [130]. Although these works mostly focused on the role of the different domains in the NO synthase activity, they also revealed that the presence of the heme domain can strongly affect the cyt *c* reductase activity and that the FMN domain from CPR could not substitute for the one originating from nNOS, even when the CaM binding domain was preserved in the chimera. On the contrary, FAD domains were perfectly exchangeable. Subsequent publications have reported a CPR-NOS chimera bearing a cyt *c* reductase activity 7-fold higher than the parental CPR [131].

More recently, the analysis of chimeric CPR combining domains from yeast and human CPR highlighted the role of interdomain interface and recognition in diflavin reductase activity and conformation [132]. The electrostatic charges at the molecular surfaces of each domain were globally preserved during the chimerogenesis since the FMN and FAD domains of nearly all diflavin reductases bear respectively negative and positive residues at their interface [43,73,133]. However, the specific interactions between precise residues in each parental CPR (human or yeast) were lost as the spatial arrangement between the two forms was not strictly respected. As a result, the internal ET rate in yeast-human chimeras decreased significantly while the hydride transfer from NADPH to FAD appeared favored compared to the parental enzyme. The slowdown of the internal ET in chimeric enzymes was therefore attributed to changes in equilibrium states of CPR induced by the perturbed recognition of the two domains in the chimeras.

Interestingly, the two opposite chimeras exhibit kinetic behaviors analogous to those of the rat nNOS mutants in which the modification of a single residue, Arg1229, located on the interface between the flavinic domains, destabilized the interdomain recognition by disrupting one specific electrostatic interaction [103]. Like chimeric CPR, this mutant had a higher FAD reduction rate and a slower internal ET rate, particularly in the CaM-free enzyme. Besides the regulatory elements specific to the NOS, the interaction surfaces between flavinic domains of diflavin reductase appear as key elements controlling the global conformation of the enzymes and therefore their ET capacity. Several hypotheses might now be proposed: (i) some displacements of the side chains of residues involved in specific interdomain interactions during the catalytic cycle could facilitate the required conformational changes, (ii) the connecting domain, besides its linking role, could also simply extend the contact surface between both catalytic domains without necessarily acting as a dynamic hinge [132].

These chimeras combining the three NOS isoforms, different CPR or the NOS and the CPR clearly demonstrate the capacity of distant diflavin reductases to exchange individual domains while totally or partially preserving their catalytic activities. CPR and NOS, and probably other diflavin reductases, are not only sharing high structural homologies. The presence of acceptable ET capacities in these different chimeras prove that, despite the architectural complexity of the regulatory elements present in NOS but absent in the other members of the family, the ET mechanisms of all diflavin reductases may involve the same structural reorganization steps. It seems then reasonable to propose that the dynamical events hypothesized from the various biochemical analyses of NOS and CPR may as well exist in all other diflavin reductases.

The above-mentioned results did not constitute a solid proof of the dynamical behavior of diflavin reductases, but they eventually cast a strong support of the existence of domains movements. In addition, the parameters controlling those movements, if partially understood in the case of the NOS, remained totally unknown for the other members of the diflavin reductase family [24,92]. Some studies suggested an influence of the NAD(P)H binding on the reductase conformation [4,105,120,121,134] or a stimulating effect of the substrate binding to the heme domain on the ET efficiency through the reductase part [105,135].

## 4. Structural Characterization of Dynamical Behaviors

### 4.1. Crystallography

**Figure 5:**
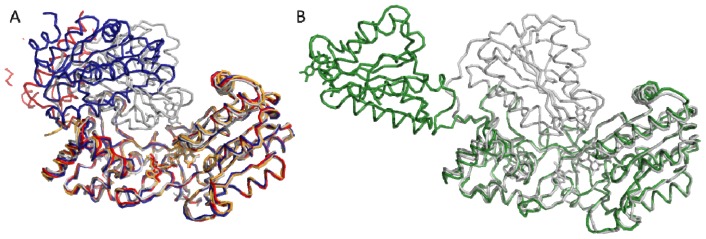
Crystallographic structures of the Δ^236^TGEE^239^ mutant [137] and yeast-human (YH) chimeric NADPH-cytochrome P450 reductase (CPR) [136]. (**A**) Structural alignment of the three conformations of the rat CPR Δ^236^TGEE^239^ mutant (PDB ID: 3ES9) and the closed form of the rat CPR (PDB ID: 1AMO). Structures of the mutant are presented in blue, red and yellow ribbons while the native closed form is shown in grey. (**B**) Structural alignment of the open conformation of the YH chimeric CPR (green ribbons, PDB ID: 3FJO) and the closed conformation of the rat CPR (grey ribbon). In this figure, all structures were superimposed on their FAD/connecting domains.

In 2009, two groups published novel crystallographic structures of mutant or chimeric CPR demonstrating the existence of alternate conformations different than the hitherto unique closed conformation [136,137]. The work of Hamdane *et al.*[137] was performed on human CPR variants containing mutations in the linker joining the FMN and connecting domains. One of these mutants, deleted from 4 residues (Δ^236^TGEE^239^, PDB ID: 3ES9), crystallized with three molecules in the crystallographic cell. As illustrated in Figure 5A, in the three conformations, the FAD and connecting domains were well-defined. However, the FMN domain was either completely invisible or only partially visible in electron density map and the precise orientation of the FMN domain could only be obtained in one copy (structure in blue in Figure 5A). The shortening of the linker had a dramatic impact on the interflavin ET, resulting in an almost complete loss of the cyt *c* reductase activity, most probably due to the incapacity of the catalytic domains to come to a position competent for interflavin ET. Consequently, the detection of this new conformation on an incompetent form of the enzyme raises the question about its existence in the course of the catalytic cycle.

On the other hand, our group published the structure of one of the abovementioned yeast-human chimeric CPR (YH) (Figure 5B, [136]). YH chimera crystallized in a widely open conformation. A structural alignment of the FAD domains of this form with the rat or yeast CPR closed forms reveals a 86 Å translation of the FMN cofactor and a rotation of almost 180° of the FMN domain itself. YH chimeric enzyme has an ET capacity of only 10% of the native CPR (measured with cyt *c*), but nevertheless remained capable of reducing other artificial and natural acceptors such as ferricyanide and P450-3A4 with a complete conservation of the electron pathway. Interestingly, the backbone of YH is almost completely conserved (RMSD 0.9 Å and 0.46 Å for the FAD and FMN domains respectively) except for the linker domain for which only a limited number (3) of residues have their Psi and Phi angles completely changed. An analysis of the interdomain interface of YH CPR [132] also revealed that the FMN domain is still making contacts with the connecting domain.

The two crystallographic structures demonstrated for the first time that open conformations could be obtained using mutant CPR. The second important information stemming from this result is that the structural reorganization of domains was quite conservative at the domain level: no massive backbone deviations within individual domains could be detected in both open forms and the sole major structural changes that could be detected concerned the general domains organization and positioning. Evidently, the intra- and intersubunit ET would then imply a succession of closing and opening mechanisms leading to the closed and open forms during the catalytic cycle of diflavin reductases. However, it is important to keep in mind that both crystallographic structures of the open forms were obtained with mutant CPR and hence do not necessarily represent open forms of the native enzymes.

### 4.2. SAXS and NMR Experiments

Early NMR experiments on MSR identified the residues at the surface of a flavodoxin that are involved in the binding of a flavodoxin reductase and of the methionine synthase [138]. This study also revealed that both physiological partners bind to unique overlapping sites on the flavodoxin, precluding the formation of a ternary complex. The authors had then inferred by analogy that the flavodoxin-like domains (FMN domains) of the diflavin reductases should form mutually exclusive complexes with their electron-donating and -accepting partners and that the interconversion between these two exclusive complexes required conformational changes.

In addition, the SAXS analysis of a human CPR mutant demonstrated that the stiffening of the linker by polyproline mutations not only induced a 50-fold decrease of the interflavin ET rate and strongly affected cyt *c* reductase activity but also changed the diffusional motion of the domains [122]. The molecular envelope of the mutant showed a restriction of the spatial volume sampled by the structural domains in solution, as compared to the elongated arrangement adopted by the domains in the native CPR. Moreover, the SAXS data also indicated that conformational changes could be affected by ligand binding, such as NADP^+^. The authors suggested that the conformational sampling required for ET and more precisely the dynamics of the highly mobile FMN domain had been severely limited in the polyproline mutant.

The coupling of SAXS and NMR spectroscopy allowed a more precise characterization of domain motion in human CPR [139]. The SAXS scattering curve obtained on the CPR was in disagreement with the closed conformation of the CPR and much better curve reproduction could be obtained by the introduction of an additional conformation with a more elongated shape. The comparison of the NMR spectra of the FMN domain either isolated or in the context of human CPR further revealed chemical shifts differences for residues involved at the interdomain interface in the closed conformation, in agreement with a significantly populated closed conformation in solution. In addition chemical shift variations were also detected in the *C*-terminal helix of the FMN domain. Since this helix is highly solvent-exposed in the closed form of CPR, the authors hypothesized that this helix might be involved in the interdomain interface in the elongated conformation observed by SAXS. Based on these data, Ellis *et al.* concluded that CPR in solution could be described using a two-state conformational equilibrium corresponding to the already known closed form of the enzyme and a novel, open form. The oxidized CPR envelop measured during the SAXS experiments corresponded to a 50:50 mixture of these two conformations.

SAXS experiments further demonstrated that the conformational equilibrium of the CPR changes upon its redox state [139]. Calculations of molecular envelopes allowed the authors to postulate that the oxidized CPR presents on average a more extended shape than the closed forms (but less extended than the open crystallographic structures above-mentioned [136,137]) while the 4-electron reduced enzyme exhibits a more or less spherical envelop reasonably consistent with the closed crystallographic structures [49,80] suggesting that the fully reduced CPR adopts a predominantly closed conformation (85:15).

Interestingly, the 2-electron NADPH-reduced CPR showed an increase in the proportion of the compact form in comparison with the oxidized enzyme but not to the same extent as the fully reduced enzyme. NADP^+^ coenzyme binding alone, without any change in the redox state of the enzyme, also produced significant changes in the conformational equilibrium with a shift in population in favor of the closed form. Finally, the 4-electron reduction and NADP^+^ coenzyme binding was required to see the complete closure. The role of ligand binding in the CPR conformational switches had long been postulated [4,120] and was then more precisely described.

Lastly, our groups revisited the solution properties of CPR by NMR [140]. This large protein (70 kDa) represented a challenge for this technique, nevertheless we managed to assign the majority (60%) of the backbone resonances of the oxidized CPR. Using ^15^N relaxation data we have demonstrated that the CPR behaves as a rigid body of the same size as the crystal closed structure and that no relative motion between the two domains was detectable. In addition, there was also an excellent correlation between experimental residual dipolar coupling (RDC) values and predicted RDC values using the closed structure. This NMR study unambiguously proved that the CPR adopts the same conformation in solution and in the crystal environment. Interestingly, we also showed the high flexibility of the linker region between the FMN and connecting domain in the nanosecond timescale. The CPR can thus be described as two beads (FAD and FMN domains) forming a stable interface bridged by a flexible string. Our results are in sharp contrast with the previous SAXS/NMR analysis. This might be explained by slightly different (but yet unidentified) experimental conditions. Since the CPR enzyme used for the NMR study is fully active, we postulated that the opening of the structure required in the course of the reaction must be provoked by some (yet unknown) molecular trigger such as substrate binding or flavin reduction.

### 4.3. ELDOR, Mass and Fluorescence Spectroscopy

Analysis of CPR dynamics using electron-electron double resonance (ELDOR) allowed a fine description of the conformational sampling of human CPR in the di-semiquinone state (one electron reduced FMN and one electron reduced FAD) [141]. The authors emphasized the importance of a continuum of conformational states across the energy landscape rather than the discrete closed and open forms described by crystallography, SAXS or NMR spectroscopy. They could confirm that the closed states were induced by nucleotide (NADP^+^) binding while open states are favored in the absence of the bound coenzyme. However, ELDOR spectroscopy describes conformational distributions exhibited by the proteins just before their internal motions are quenched by rapid freezing at 80 K. Therefore, the observed distance distribution may not represent distances between points on heavy atoms, but rather a weighted mean of the unpaired electron spin density distribution over the flavosemiquinones in absence of domain mobility. Nonetheless, the distance variations observed upon NADP^+^ binding and salt effects were in agreement with the SAXS experiments [139].

Averaged distance values measured by PELDOR in NADP^+^-bound CPR are in good agreement with the observed distances in the available closed crystal structures (15.4 Å compared to 13.4 Å between the two N5 atoms of FAD and FMN, [49]) and with the less open conformation of the deleted CPR mutant crystallized by Hamdane *et al*. (31.6 Å, [137]). The large distances observed in the mutant CPR (deleted in the linker or chimeric YH) are not in agreement with the ones calculated with ELDOR spectroscopy, yet we should bear in mind that CPR in the crystallographic state or in the ELDOR conditions do not present the same oxidation state. Nonetheless, the ELDOR data suggest that the di-semiquinone human CPR is present, in solution, as a distribution of multiple extended open and compact closed conformations. The authors also investigated the influence of pressure on ET rate. They correlated high pressure values with higher interflavin ET rates. This again seems to indicate more compact enzyme conformations since the interflavin ET is postulated to be conformationally gated [121].

Another set of evidences of dynamical behavior came from FRET experiments performed by the group of Scrutton [142]. Human wild-type CPR was labeled on surface accessible cysteinyl residues by two compatible fluorophores and the FRET intensity was measured at different oxidations stages (oxidized, two electrons and four electrons reduced CPR). Again, the authors clearly saw a change in the FRET signal when the NADP^+^ nucleotide binds to CPR, irrespective of its oxidation state. Exact evaluation of the distances between fluorophores could not be evaluated unambiguously but seemed to indicate a closure upon reduction and NADP^+^ binding, in agreement with the previous SAXS [139] and ELDOR [141] studies. Furthermore, the fact that three surface cysteinyl residues were partially labeled (one of them being a completely conserved, catalytic residue close to the nucleotide binding pocket) raised some possible doubts about the exact movement (opening or closing) observed. In 2011, Jenner *et al*. used ion mobility spectrometry in combination with electrospray ionization and mass spectrometry to study the dynamic equilibrium in oxidized CPR [143]. They found that two major conformations of CPR, closed and extended, coexist in the gas phase and that this equilibrium is affected by the ionic strength and changes in the redox states. Taken together, these experiments added further evidences of the dynamical behavior of CPR.

In summary, there is now a wealth of experimental data using far-related techniques (SAXS, NMR, ELDOR, MS) that converge towards the existence of a wide conformational space that the human CPR may sample in solution. Furthermore, the energy landscape appears to be largely influenced by substrate binding or flavin reduction, which may reveal the complex domain rearrangement during the catalytic reaction. However, despite the huge recent progress, a thorough characterization of the CPR is still lacking that may include a better understanding of the structure and dynamics of the extended conformation and its interactions with partners and a more precise description of the time-dependent conformational change during the catalytic cycle. Similarly, the concepts developed for the CPR will have to be tested for the other related proteins.

### 4.4 Kinetic Models

**Figure 6:**
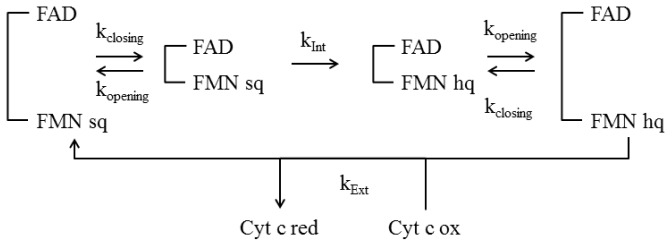
Kinetic model showing the interconversion process during ET from NOS to c yt *c*. (adapted from [55]). *k*_opening_ and *k*_closing_ are the rate constants for the interconversion reactions between the closed (shielded) and open (deshielded) forms. *k*_Int_ and *k*_Ext_ are the rate constants for internal ET (between flavins) and external ET (to cyt *c*) respectively. Sq and hq refer to the semiquinone and hydroquinone states of FMN.

Different models have been proposed in the past to describe the successive electrons transfers [4,144], however they do not include the conformational state of the enzyme. It is evidently mandatory that all the dynamical models that were collected in the last few years find a theoretical support able to describe how the opening and closing rates can be related to the overall kinetic cycle of diflavin reductases. More recently, a kinetic model has been proposed in this direction and aims at linking variation of the interconversion rates from the shielded (closed) to the deshielded (open) conformation (and reverse) to the existing ET kinetic data on various NOS [145]. In this kinetic model (see Figure 6), the authors assume that the protein samples the two conformational states (shielded and deshielded) with similar rate constants, irrespective to cofactor binding or reduction. They also suppose that the shielded state is the only one competent for interflavin ET with a rate constant *k*_Int_ and that the deshielded state is the only one competent for external ET (rate constant *k*_Ext_). Using such a simple kinetic model linking for the first time conformational sampling and electron transfer they have been able to reproduce some experimental data on NOS. For example, this model explains how the slow conformational equilibrium, and hence the supposed conformational motion of the FMN domain, directly controls the intensity of the slow and fast phase of FAD reduction and the electron flux to cyt *c*[145]. In addition, when the closing and opening phenomena are supposed to be the rate-limiting steps of the cycle, the model predicts a bell shape for the steady-state reduction rate *versus* the fraction of (de)shielded state which nicely fits experimental data obtained on various nNOS forms [55]. However, the model is still far too simplistic and for example, the NADPH binding, hydride transfer, interflavin ET, and NADP^+^ release steps are all integrated in a single step (*k*_Int_). In addition, there are experimental evidences for significant effects of flavin reduction and cofactor binding on the conformational equilibrium, which are not taken into account in the model. It seems then possible to improve the kinetic model at the expense of additional data collection for validating the increased number of elementary steps. There is no doubt that future investigations on the coupling between conformations sampling and kinetic properties of diflavin reductase will provide invaluable clues on the microscopic behavior of the enzyme at work.

## 5. Conclusions and Future Perspectives

Starting from the first published CPR structure, in 1997, the scientific community has tracked avidly alternate architectures of diflavin reductases that would be compatible with ET to external acceptors. We have now plenty of evidences that, at least for CPR and NOS, reorientation of the FMN and FAD domains occurs at some stage during the electronic cycles. The alternative model described with the yeast CPR and involving the rotation of the FMN cofactor instead of the FMN domain seems not to fit with the very recent evidences of domain movements in human and rat CPR. As of today, the model involving conformational changes is really favored. The fact that several structural organizations were characterized by different methods seems to indicate that diflavin reductases may exist as a mixture of some discrete or continuous conformations in equilibrium. This equilibrium appears to be easily disturbed by mild change in working conditions, at least for the oxidized form of the CPR, suggesting that great care should be taken when comparing structural studies carried out in different laboratories. As a result the published crystallographic structures probably represent the most populated conformations depending on the enzyme considered (wild-type or mutant). Even if these crystallographic structures do not correspond to highly populated conformers in solution, they still demonstrate that reorientation of the catalytic domains occur with almost no change within the backbone of the domains themselves. This is also true for the connecting domain of CPR for which no specific role has yet been assigned. This connecting domain, as well as the various regulatory domains of NOS or MSR having more complex catalytic regulation, probably plays a very important role in the sensing mechanisms and in the control of the domains structural reorganization. The surfaces of these regulatory domains, being in direct contact with both catalytic domains, are probably the place where this information exchange takes place.

That being said, we still lack information on the possible sensing mechanisms and how they are transmitted within the enzyme prior any structural reorganization. The involvement of cofactors and redox potential were described. Whether these factors are involved in changing existing conformational equilibria (by selectively binding to and lowering the energy of extended conformations) or directly impact structural points that could trigger domain reorganization is still under debate and will probably be the subject of intense and ardent research in the next years. Another open question concerns the role of the physiological acceptors in the control of these movements. From the simpler model of CPR to the more evolved NOS, the regulation of ET in diflavin reductases is of crucial importance for the enzyme and most probably to avoid electron leakage to oxygen and the consequent production of toxic ROS. It is likely that the presence or absence of these acceptors drive the equilibrium between the various conformations and therefore may also constitute some of the necessary triggers. Such propositions will probably constitute future research directions in the field of diflavin reductase mechanisms.

## Figures and Tables

**Table 1:** NOS chimeras composed of domains from the eNOS (light orange), the nNOS (light green) and the iNOS (light blue gray). The description of the domains in the chimeras refers to the color of each parental isoform. Heme: domain oxygenase, CamBD: CaM binding domain, FMN: FMN domain and FAD: FAD-connecting domains. Activities are expressed as a percentage relative to the activity of iNOS which is the most active of the 3 isoenzymes. Ox refers to the oxidase domain, Red to the diflavin domain.

Diflavin reductase	Domains				% NOS activity	References
	Heme	CamBD	FMN	FAD		
eNOS					10	
nNOS					60	
iNOS					100	
iNOS-nCamBD					100	[25]
nNOS-iCamBD					30	[25]
iOx-nRed					100	[25]
nOx-iRed					0	[25]
eOx-nRed					60	[129]
nOx-eRed					20	[129]
eNOS-nCamBD					40	[110]

**Table 2:** Cyt *c* and ferricyanide reductase activities of nNOS-CPR chimera. Domains from the nNOS and the rat CPR are indicated by blue and red colors respectively. The description of the domains in the chimeras refers to the color of parental reductase. Heme: domain oxygenase, CamBD: CaM binding domain, FMN: FMN domain and FAD: FAD-connecting domains. Activities are expressed as a percentage relative to the activity of nNOS [130].

Diflavin reductase	Domains				% activity		
	
	Heme	CamBD	FMN	FAD	NOS	cyt *c*	ferricyanide
nNOS					100	100	100
CPR						75	20
Chimera 1					>100	50	50
Chimera 2					≈0	15	>100
Chimera3						>100	50
